# FGF-1 and FGF-2 modulate the E-cadherin/catenin system in pancreatic adenocarcinoma cell lines

**DOI:** 10.1054/bjoc.2001.1813

**Published:** 2001-06

**Authors:** I EI-Hariry, M Pignatelli, N R Lemoine

**Affiliations:** Imperial Cancer Research Fund Molecular Oncology Unit; Department of Histopathology, Imperial College School of Medicine, Hammersmith Campus, Du Cane Road, London W12 0NN, UK

**Keywords:** E-cadherin, catenins, FGF, FGFR, pancreatic adenocarcinoma

## Abstract

Fibroblast growth factors (FGFs) and fibroblast growth factor receptors (FGFRs) have been increasingly recognized to play an important role in the pathobiology of pancreatic malignancy. We have investigated the effects of FGF-1 and FGF-2 on the behaviour and adhesion properties of human pancreatic adenocarcinoma cell lines (BxPc3, T3M4 and HPAF) that were previously characterised for the expression of FGFRs. Here we show that exposure to FGF-1 and FGF-2 leads to significant and dose-dependent increase in E-cadherin-dependent cell-cell adhesion, tubular differentiation, and a reduced capacity to invade collagen gels. FGF stimulation produces phosphorylation of E-cadherin and β-catenin on tyrosine residues, as well as increased E-cadherin localisation to the cytoplasmic membrane and association with FGFR1 demonstrable by coimmunoprecipitation. These results demonstrate that FGF-1 and FGF-2 may be involved in the regulation of cell adhesion, differentiation and invasion of pancreatic cancer. © Cancer Research Campaign http://www.bjcancer.com


*British Journal of Cancer* (2001) **84**(12), 1656–1663
© 2001 Cancer Research Campaign
doi: 10.1054/ bjoc.2001.1813, available online at http://www.idealibrary.com on IDEAL®

http://www.bjcancer.com

### FGF-1 and FGF-2 modulate the E-cadherin/catenin system in pancreatic adenocarcinoma cell lines

**I El-Hariry[1], M Pignatelli[2] and NR Lemoine[1]**

[1]Imperial Cancer Research Fund Molecular Oncology Unit; [2]Department of Histopathology, Imperial College School of Medicine, Hammersmith Campus, Du Cane Road, London W12 0NN, UK

**Summary** Fibroblast growth factors (FGFs) and fibroblast growth factor receptors (FGFRs) have been increasingly recognized to play an important role in the pathobiology of pancreatic malignancy. We have investigated the effects of FGF-1 and FGF-2 on the behaviour and adhesion properties of human pancreatic adenocarcinoma cell lines (BxPc3, T3M4 and HPAF) that were previously characterised for the expression of FGFRs. Here we show that exposure to FGF-1 and FGF-2 leads to significant and dose-dependent increase in E-cadherin-dependent cell-cell adhesion, tubular differentiation, and a reduced capacity to invade collagen gels. FGF stimulation produces phosphorylation of E-cadherin and β-catenin on tyrosine residues, as well as increased E-cadherin localisation to the cytoplasmic membrane and association with FGFR1 demonstrable by coimmunoprecipitation. These results demonstrate that FGF-1 and FGF-2 may be involved in the regulation of cell adhesion, differentiation and invasion of pancreatic cancer. © Cancer Research Campaign http://www.bjcancer.com

**Keywords**: E-cadherin; catenins; FGF; FGFR; pancreatic adenocarcinoma

Changes in cell adhesion, regulated by environmental signals such as growth factors, appear to be necessary for dynamic cellular movement and maintenance of tissue patterning. E-cadherin is well-established as playing a suppressive role in the progression of malignant disease (Takeichi, 1991). Selective reduction or loss of E-cadherin is correlated not only with dedifferentiation, but also with the invasive phenotype in vitro and in vivo (Birchmeier et al, 1993; Pignatelli et al, 1992). This has been reported in pancreatic carcinoma, where loss of membranous E-cadherin correlated with high grade and advanced tumour stage (Pignatelli et al, 1994). Furthermore, the role of E-cadherin in tumour progression has been explored using specific inhibitors and gene transfer experiments. Transfection of E-cadherin-negative colorectal carcinoma cells with E-cadherin cDNA restores not only their capacity for intercellular adhesion but also results in reversal of the invasive phenotype (Liu et al, 1993).

A growing body of evidence suggests a role for the FGF/FGFR system in the modulation of cell adhesion and cell migration. It has been demonstrated that neurite outgrowth responses stimulated by cell adhesion molecules (CAMs) are mediated via FGFR, probably as a direct consequence of an interaction via the CAM homology domain (Doherty et al, 1995). FGFR-CAM interaction was further substantiated by identifying and characterizing a CAM homology domain (CHD) within the extracellular domains of FGFR, with similarity to regions found in L1, NCAM (VASE motif) (Williams et al, 1994) and N-cadherin (HAV motif) (Byers et al, 1992). These regions in CAMs have been implicated in the mediation of homophilic interactions between cadherin molecules. Subsequently, anti-FGFR antibodies and CHD peptides were both shown to inhibit neurite outgrowth responses to cell adhesion molecules and FGF-2 (Doherty et al, 1995).

Finally, indirect evidence comes from observations that the expression levels and/or functions of growth factor receptor tyrosine kinases (RTK) and E-cadherin may be co-ordinated. In this context, β-catenin has been shown to associate with at least two receptor tyrosine kinases: epidermal growth factor receptor (EGFR) (Hoschuetzky et al, 1994) and c-erB-2 (Kanai et al, 1995). Ligand-induced activation of these receptors also results in increased tyrosine phosphorylation of β-catenin and subsequent impaired cadherin/catenin function by a mechanism that is still poorly understood.

We have examined the possible involvement of E-cadherin in determining the invasive phenotype of pancreatic carcinomas and investigated whether E-cadherin functions can be modulated by stimulation with exogenous FGF-1 and FGF-2 in a panel of human pancreatic adenocarcinoma cell lines that express different combinations of FGFRs.

### MATERIALS AND METHODS

### Cell lines

Human pancreatic adenocarcinoma cell lines that express various combinations of FGFRs (Leung et al, 1994) and exhibit different grades of differentiation were used. The BxPc3 cell line (American Type of Culture Collection (ATCC), Rockville, Maryland) is a moderately to well differentiated cell line derived from a primary tumour (Tan et al, 1986) and expresses FGFR-1; the T3M4 cell line is a moderately differentiated cell line, which was derived from a lymph node metastasis (Okabe et al, 1983) and expresses FGFR-3; and the HPAF cell line which is a moderately to poorly differentiated cell line that was derived from ascitic fluid (Metzgar et al, 1982) and is FGFR-3 and FGFR-4 positive. All cell lines were maintained in standard medium.

*Received 7 September 2000*
*Revised 5 March 2001*
*Accepted 6 March 2001*

*Correspondence to:* NR Lemoine

### Growth factors and antibodies

Human recombinant FGF-1 and FGF-2 (Upstate Biotechnology, Lake Placid, NY, USA) were used for stimulation of the cell lines at various concentrations between 1 ng/ml and 50 ng/ml. Heparin was added at 10 μg/ml and 1 μg/ml with FGF-1 and FGF-2, respectively, as shown to be suitable for FGFR activation by FGFs in this tissue type (Leung et al, 1994).

The mouse monoclonal anti-human E-cadherin antibody, HECD-1, was kindly provided by Prof. Takeichi (Kyoto University, Kyoto, Japan). Mouse monoclonal anti-human E-cadherin, α-, β-and γ-catenin antibodies were purchased from Transduction Laboratories, Exeter, UK. The monoclonal anti-Ep-CAM (AUA-1) and anti-CEA (PR3B10) antibodies were kindly provided by Sir Walter Bodmer (ICRF). The anti-FGFR antibodies used in the study were anti-FGFR-1 antibodies (8E10 (Prizm Pharmaceuticals) and VBS6 (Santa Cruz)). Anti-phosphotyrosine antibody 4G10 was purchased from Upstate Biotechnology.

### Cell–cell adhesion assay

Cells were grown to 90% confluence, washed twice in PBS, and subsequently treated with 2 mM EDTA for 10 min at 37˚C. Detached cells were washed once with RPMI medium and were passed through Pasteur pipettes several times to obtain single cells. Cells were then re-suspended in either $Ca^{2+}$-free PBS/0.8% FCS or RPMI/0.8% FCS (controls). In FGF stimulation experiments, FGF-1 or FGF-2 was added to the medium at concentrations of 1, 5, 10, 20, and 50ng/ml at the time of initiation of the assay. Cells were then inoculated at $5\times10^5$ cells/ml into 24-well plates (500μl/well) that had been coated overnight with 1% (w/v) bovine serum albumin in PBS to prevent non-specific cell adhesion to wells. Cells were allowed to aggregate for 1 h at 37˚C on a gyratory shaker with constant rotation at 90 rpm. Cell aggregation was terminated by the addition of 4% (w/v) glutaraldehyde fixative to individual wells at 0, 15, 30, 45 or 60 min. Aliquots were taken at each time point and the number of single cells was then determined with a Coulter counter (Coulter Electronics, Inc.). Cell-cell adhesion was assessed by the cell aggregation index (Nt/NO), where Nt is the number of single cells after the incubation time, and NO is the number of single cells at the initiation of the assay.

### Blocking of E-cadherin-mediated cell adhesion

Experiments were carried out as above except that cell suspensions were incubated with 10 μg/ml anti-E-cadherin (HECD-1) antibody for 30 min before the initiation of the assay. The extent of inhibition of cell adhesion was represented as percent inhibition (Ni/Nc X 100%), where Ni is the number of single cells in the presence of anti-E-cadherin antibody and Nc is number of single cells in the absence of the antibody (control).

### In vitro invasion system in collagen gel

Collagen gels were prepared by mixing eight volumes of ice-cold collagen type I stock solution (Vitrogen 100, Imperial Laboratories, UK) with one volume of 10X DMEM containing phenol red indicator and neutralised with 0.1 M NaOH. Aliquots (1 ml) were added into 35 mm tissue culture dishes and incubated for 1 h at 37˚C in a 10% $CO_2$ incubator. After gelation, $1X10^5$ cells re-suspended in 1 ml of RPMI/1% FCS alone (control) or supplemented with FGF-1 or FGF-2 (1, 5, 10, or 20 ng/ml) were seeded onto the gel. Cells were re-fed every 3 days for 10 days with the standard medium alone (control) or supplemented with FGF, and growth was assessed daily using a phase contrast microscope. Gels were finally fixed in 10% formaldehyde overnight, paraffin embedded, serially sectioned in vertical orientation (5 μm sections), stained with haematoxylin and eosin and photographed. The number of cells that invaded the gel in 10 randomly selected graticule areas from duplicate cultures was determined. The degree of invasion was represented by the invasion index (Nv/Nc), where Nv is the number of invading cells in stimulated cultures and Nc is the number of invading cells in control cultures. In some experiments, serial dilutions of monoclonal anti-E-cadherin (HECD-1), anti-CEA and anti-EP-CAM antibodies were added to the culture medium for five consecutive days. Purified mouse immunoglobulins (50μg/ml) were also used as control in these experiments.

### Morphogenic assay in 3-D collagen gels

Experiments were carried out as above except that 1ml of the single cell suspension was mixed with 10 volumes of the neutralized vitrogen solution to yield a final concentration of $1\times10^5$ cells/ml, and the solution was allowed to gel at 37˚C. Gels were then overlaid with the standard medium alone (control) or medium supplemented with FGF-1 or FGF-2 (10 ng/ml), that was replaced every 4 days for 21 days. Plates were checked daily for the appearance of glandular structures using a phase-contrast microscope.

### Immunohistochemistry

Sections prepared from the morphogenic assay were deparaffinized in xylene and rehydrated in graded alcohols. An avidin-biotin peroxidase method was applied. Tris-buffered saline (TBS) was used in all washing steps. Non-specific binding was blocked with normal rabbit serum, and sections were probed with optimally diluted primary antibody for 2 h, washed, and then incubated with biotinylated rabbit anti-mouse antibody (Dakopatts, Denmark; 1:200) for 30 min, followed by freshly prepared avidin-biotin-peroxidase complex (ABC) for 30 min. Sections were developed in 0.05% (w/v) diaminobenzidine (DAB) and 0.03% (v/v) hydrogen peroxide in PBS, counterstained in haematoxylin and mounted. As a negative control, the primary antibody was replaced by TBS. The sections were assessed for the subcellular localisation of E-cadherin and catenins (membranous, cytoplasmic, mixed membranous and cytoplasmic). The staining intensity of E-cadherin and catenins were also graded semiquantitatively using an arbitrary scale of intensity: – (no expression); + (increased expression); and +/– (equivocal expression).

### Immunoprecipitation and immunoblotting

These experiments were performed as previously described (El-Hariry et al, 1999). Briefly, cell lysates (soluble fraction) were precleared and incubated with anti-E-cadherin, anti-α-, anti-β-, and anti-γ-catenin antibodies overnight at 4˚C with rotation. Immune-complexes were precipitated with 50 μl/ml protein G-sepharose, and the immunoprecipitates were resolved by SDS-PAGE and transferred onto nitrocellulose membranes (Millipore, Herts, UK) for immunoblotting. After non-specific binding was blocked, membranes were probed overnight with:

© 2001 Cancer Research Campaign

(i) anti-E-cadherin (1 μg/ml); (ii) anti-β-catenin, 1 μg/ml; (iii) anti-α-catenin, 4 μg/ml and (iv) anti-γ-catenin, 1 μg/ml. Membranes were incubated with peroxidase-conjugated secondary anti-mouse antibodies (Dako Ltd, Bucks, UK; 0.5 μg/ml) for 2 h at room temperature, washed, and then were reacted with enhanced chemiluminescence reagent (ECL; Amersham Life Sciences, Bucks, UK) and exposed to Hyperfilm-MP film (Amersham Life Sciences, Bucks, UK). Cell lysates immunoprecipitated with IgG were used as a control for the antibody specificity.

The protein levels were assessed by densitometric analysis using ImageQuant image analysis software package (Molecular Dynamics, Inc, Bucks, UK). Results were presented in arbitrary units of the integrated optical density (OD). All experiments were repeated twice.

### Statistical analysis

Where the effect of either FGF-1 or FGF-2 was compared to the control samples, the significance of differences between groups was calculated by the Student's two-tailed *t*-test. The mean and the standard deviation (SD) were calculated from at least three separate experiments. The effect of FGF-1 and FGF-2 on cell-cell adhesion as a function of FGF doses was computed using the analysis of variance (F-test) to compare the difference between control, FGF-1 and FGF-2. The level of significance was taken as $P < 0.05$.

### RESULTS

### Cell-cell adhesion in pancreatic cell lines

In the absence of $Ca^{2+}$, all pancreatic cell lines showed poor spontaneous aggregation, but they readily formed cell aggregates in the presence of $Ca^{2+}$. The increase in cell aggregation was small but statistically significant. This finding indicated that cell-cell adhesion was mediated by a $Ca^{2+}$-dependent mechanism. Since this aggregation was significantly blocked by the addition of the monoclonal anti-E-cadherin antibody (HECD-1), it was apparent that E-cadherin was the most likely candidate implicated in cell-cell adhesion and was functionally active in these cell lines. As additional controls, incubation with anti-CEA and anti-EP-CAM antibodies did not block cell-cell adhesion in $Ca^{2+}$-supplemented cell suspension (RPMI medium), thus giving further evidence for the specificity of E-cadherin-mediated effects.

Both FGFs significantly increased cell aggregation in BxPc3 ($P < 0.01$), T3M4 ($P < 0.001$) and HPAF cell lines ($P < 0.001$) only in the presence of $Ca^{2+}$, Figure 1(A-C). FGFs appeared to modulate the kinetics of the cell-cell adhesion. For instance, in HPAF cells, while half-maximal aggregation was not reached until 55 min in untreated cells, it occurred within ~15 min in cells treated with FGF-2 and FGF-1, Figure 1C. Furthermore, cell aggregates appeared large and coalescent in the presence of FGF-1 or FGF-2 as opposed to small and irregular aggregates in untreated cells.

The modulation of cell-cell adhesion by FGFs was E-cadherin-dependent, since anti-E-cadherin antibody inhibited the effect of FGF-1 and FGF-2 on cell-cell adhesion almost completely in the three cell lines, Figure 1D. In contrast, the modulatory effect of FGF-1 or FGF-2 was not blocked by the treatment of cell suspensions with anti-CEA and anti-EP-CAM antibodies in $Ca^{2+}$-supplemented or $Ca^{2+}$-free medium. The effect of FGF-1 and FGF-2 on cell-cell adhesion is dose-dependent in the range of 1 ng/ml to 20 ng/ml, which was statistically significant, Figure 1E.

### In vitro invasion into collagen gel

The cell lines used in the current study varied in their migratory ability through the gel matrix. Figure 2 depicts the effect of FGF-1 and FGF-2 on the invasive behaviour of these cell lines. Both BxPc3 and T3M4 cell lines grew into the gel front as solid sheets or as confluent irregular islands of cells, and exhibited a weak to moderate invasive capacity. FGF-treated cells became more cohesive and less invasive. HPAF cells assumed a pleomorphic appearance and were markedly invasive. In the presence of FGF-1 or FGF-2, cells became more cohesive than control untreated cultures and less invasive into the collagen. The effect of FGFs was demonstrated at concentrations as low as as 1 ng/ml and up to 20 ng/ml. The effect of FGF was E-cadherin-mediated, since it was abrogated by treatment with HECD-1 antibody, but not with anti-CEA, anti-EP-CAM antibodies or purified mouse IgG.

### Morphogenic assay in 3-D collagen gels

Stimulation with FGF-1 or FGF-2 did not produce any discernible effects on morphogenesis in either BxPc3 or T3M4 cells. In contrast, both FGF-1 and FGF-2 induced glandular differentiation in HPAF cells, where organised structures consisting of a single layer of cells with basally arranged nuclei around a central lumen were observed, Figure 3.

### FGFs increase tyrosine phosphorylation of E-cadherin/catenin system

In addition, tyrosine phosphorylation of E-cadherin and catenins was examined. Serum-starved cells were treated with medium alone or supplemented with either FGF-1 or FGF-2 for 15 min. Lysates were immunoprecipitated with anti-E-cadherin, anti-α-catenin, anti-β-catenin or anti-γ-catenin antibodies, separated by SDS-PAGE, and the immunoblots were probed with anti-phosphotyrosine antibody. Figure 4 depicts the tyrosine phosphorylation of E-cadherin and catenins in control and FGF-stimulated cells. Both FGF-1 and FGF-2 induced approximately 2-fold increase in the tyrosine phosphorylation of E-cadherin in BxPc3 cells (Figure 4A, lanes 2 and 3), as compared to 3-fold and 6-fold increase in HPAF cells respectively, Figure 4A, lanes 8 and 9. Similarly, both FGFs induced an approximately 2-fold increase in tyrosine phosphorylation of β-catenin in both cell lines (Figure 4C, lanes 2, 3, 8 and 9), and α-catenin in only HPAF cells, Figure 4B, lanes 8 and 9. While both FGFs produced an increase in tyrosine phosphorylation of β-catenin (Figure 4C, lane 5), they had only a marginal effect on E-cadherin, α-catenin, or γ-catenin in T3M4 cells.

### FGFs upregulate the expression of E-cadherin and catenins

Formalin-fixed, paraffin-embedded sections of the control and FGF-treated cells were stained with anti-E-cadherin, anti-α, anti-β-and anti-γ-catenin antibodies. As shown in Table 1, both FGF-1 and FGF-2 up-regulated the expression of E-cadherin and/or one or more of the catenins. The immunoreactivity of E-cadherin (Figure 5A), β-catenin (Figure 5B) and γ-catenin was restricted to membranous localisation on FGF stimulation.

 © 2001 Cancer Research Campaign

### Physical association of FGFs and E-cadherin/catenin

We next examined the possibility of a physical association between the E-cadherin/catenin and FGFR systems. E-cadherin/catenin complexes from confluent cultures of BxPc3 cells grown in standard medium were immunoprecipitated with antibodies against E-cadherin, β-catenin and FGFR-1, and the immunoblots were probed with either anti-E-cadherin or anti-FGFR-1 antibodies. As shown in Figure 6A, E-cadherin was detected as a faint band in FGFR-1 immunoprecipitates. In the reciprocal experiments, these immunoprecipitates were probed with anti-FGFR-1 antibody. As shown in Figure 6B, FGFR-1 co-migrated with E-cadherin immunoprecipitates as a fainter signal (lane 2), and with β-catenin (lane 3). In similar experiments, no association was detected between FGFR-1 and either α-catenin or γ-catenin (data not shown). These findings suggest that the association of FGFR-1 with the E-cadherin/catenin system in BxPc3 cells may primarily involve β-catenin.

In the second set of experiments, BxPc3 cells were serum-starved, and then stimulated with FGF-2 (50 ng/ml) for 24 h. Cell extracts were immunoprecipitated with anti-E-cadherin and anti-FGFR-1 antibodies, and the immunoblots were probed with anti-E-cadherin antibody. As shown in Figure 6C, E-cadherin was detected in FGFR-1 immunoprecipitates from both FGF-treated and untreated cell extracts (lanes 1 and 2 respectively). Furthermore, E-cadherin was markedly increased in FGF-treated cells (lane 3) as compared to control (lane 4).

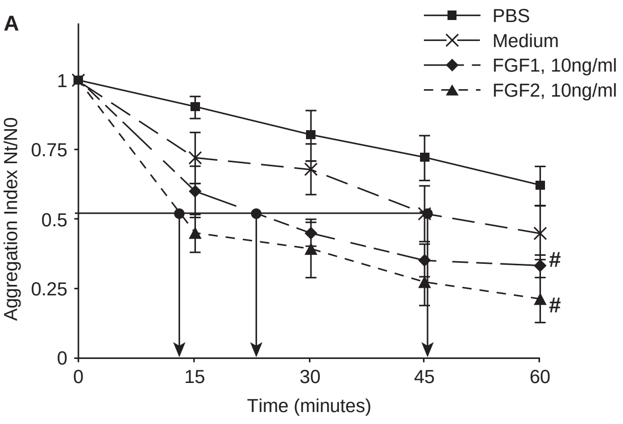

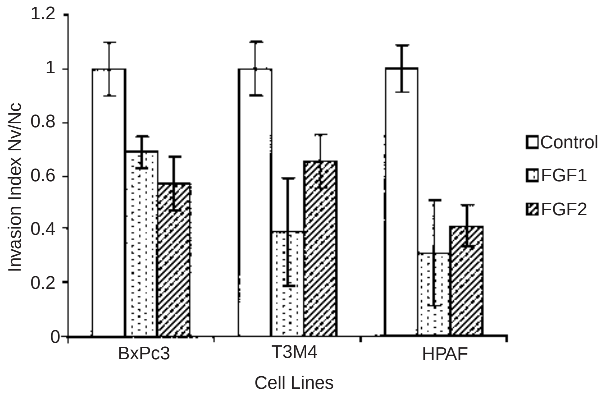

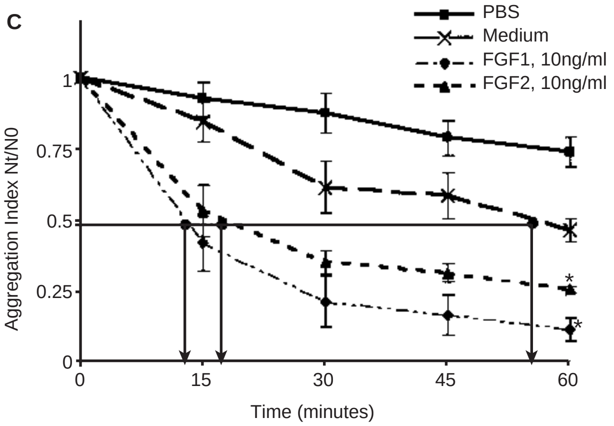

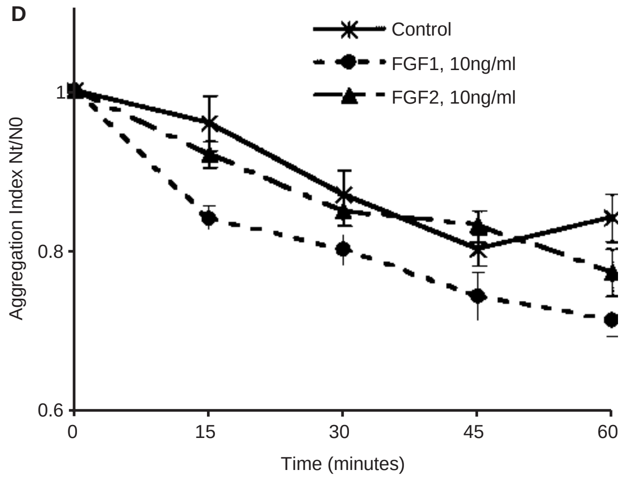

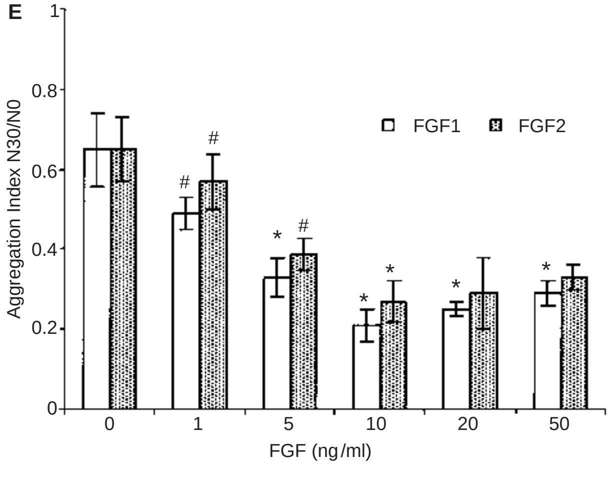

**Figure 1** Cell–cell adhesion assay. (**A–C**) Effect of FGF-1 and FGF-2 on cell-cell adhesion in BxPc3 (**A**), T3M4 (**B**) and HPAF cells (**C**). Cells were harvested from confluent monolayer cultures by EDTA and suspended in PBS ($Ca^{2+}$-free), RPMI medium (2 mM $Ca^{2+}$) or treated with 10 ng/ml FGF-1 and FGF-2 (added to RPM1/0.8% FCS). The accumulation of cell aggregates was determined at times 0, 15, 30, 45 and 60 min using a Coulter counter. The time to half-maximal aggregation is indicated by arrows. (**D**) Blocking of cell-cell adhesion on addition of anti-E-cadherin (HECD-1) antibody to FGF-treated or untreated (control) T3M4 cells 30 min before initiation of the assay. (**E**) Effect of FGF-1 and FGF-2 on cell-cell adhesion as a function of dose (1, 5, 10, 20 and 50 ng/ml) in HPAF cells. The accumulation of aggregates was determined after 30 min. #, $P < 0.01$; *, $P < 0.001$. All experiments were carried out in duplicate and the results represent the means ± SD of at least six separate experiments

© 2001 Cancer Research Campaign

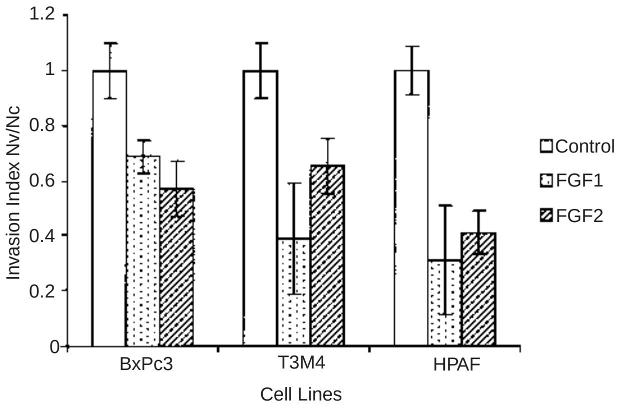

**Figure 2** Effect of FGF-1 and FGF-2 on the invasive behaviour into collagen gel. Collagen gels were freshly prepared (see material and methods), and $1 \times 10^5$ cells re-suspended in 1 ml of RPM1/1% FCS (control) or supplemented with FGF-1 or FGF-2 (1, 5, 10, 20 ng/ml) were seeded onto the gel. The assay was maintained for 10 days. Gels were then fixed in 10% formaldehyde overnight, paraffin embedded, serially sectioned, and stained with haematoxylin and eosin. The degree of invasion was represented by the invasion index (Nv/Nc), where Nv is the number of invading cells in stimulated cultures and Nc is the number of invading cells in control culture. These results represent the mean ± SD of three independent experiments

### DISCUSSION

Increasing evidence suggests that changes in the expression levels and/or functional state of RTK and E-cadherin may be related cellular events that are associated with tumour progression (Hoschuetzky et al, 1994; Kanai et al, 1995). The potential involvement of the FGF/FGFR system in E-cadherin-mediated functions is less well explored. FGF/FGFR interaction was reported to modulate the neurite outgrowth function of NCAM (Williams et al, 1994). Similar to other RTKs, signals mediated by FGF/FGFR induce a multitude of cellular activities from differentiation to proliferation. These observations prompted us to investigate: (a) the possible involvement of FGF/FGFR in the modulation of the functional activities of E-cadherin/catenin system, and (b) possible molecular interactions between FGFR and the E-cadherin/catenin complex.

Cell lines used in this study displayed adhesive ability that was $Ca^{2+}$-dependent and E-cadherin-mediated. Both FGFs modulated cell-cell adhesion from a sluggish to an accelerated rate and from a weak to a strong adhesion state. The rapidity of cell adhesion suggested that FGFs led to a direct activation of E-cadherin complexes, rather than modulation of expression of E-cadherin. A possible explanation is that FGFs lead to a rapid relocalisation of E-cadherin to the plasma membrane and subsequent recruitment of E-cadherin and catenins into complexes. This view is supported by our observations that the components of the E-cadherin/catenin complex partition into plasma-bound and cytosolic pools in these cell lines (El-Hariry et al, 1999). Additionally, the formation of large compact aggregates in response to FGFs may suggest increased coupling of the E-cadherin/catenin system to the actin cytoskeleton (El-Hariry, unpublished data) and subsequent increase in E-cadherin adhesive ability.

These findings add further support to the observations that various growth factors exert distinct effects on the E-cadherin/-catenin system. While the adhesive function of E-cadherin/catenin complex can be restored by insulin-like growth factor I (IGF-I) in human breast cancer MCF-7/6 cells (Bracke et al, 1993), other growth factors such as EGF were shown to reduce E-cadherin-mediated cell-cell adhesion and perturb its association with cytoskeletal proteins (Hazan and Norton, 1998).

We also demonstrated that the pancreatic cell lines differed in their invasive ability, which can be modulated by FGFs. The invasive ability of these cell lines was not suppressed completely in the presence of FGFs, which emphasizes the fact that other molecular mechanisms play a role in the invasion process. Modulation of E-cadherin function was also observed in the presence of insulin-like growth factor I (IGF-I) (Bracke et al 1991), and tamoxifen (Bracke et al, 1994). Taken together, FGFs could be added to the growing list of factors that lead to the modification of E-cadherin functions.

An interesting finding in the present study was the induction of differentiation in the HPAF cell line. FGFs are known for their pleiotropic effects in vivo that include mitogenic, migratory and differentiation responses (Roghani and Moscatelli, 1992). FGFs may induce a morphogenic process through augmentation of cell-cell and/or cell-stromal interactions. In this regard, FGF-2 has been shown not only to upregulate many integrin receptors in vitro and in vivo, but also potentiate their adhesive and signalling functions (Kinoshita et al, 1993; Miyamoto et al, 1996). Interestingly, cross-talk has recently been reported between E-cadherin and $\alpha 2\beta 1$ integrin (Pignatelli et al, 1997); the latter plays an essential role in epithelial renewal and promotes terminal differentiation of cultured keratinocytes (Watt and Hertle, 1994). The cell surface

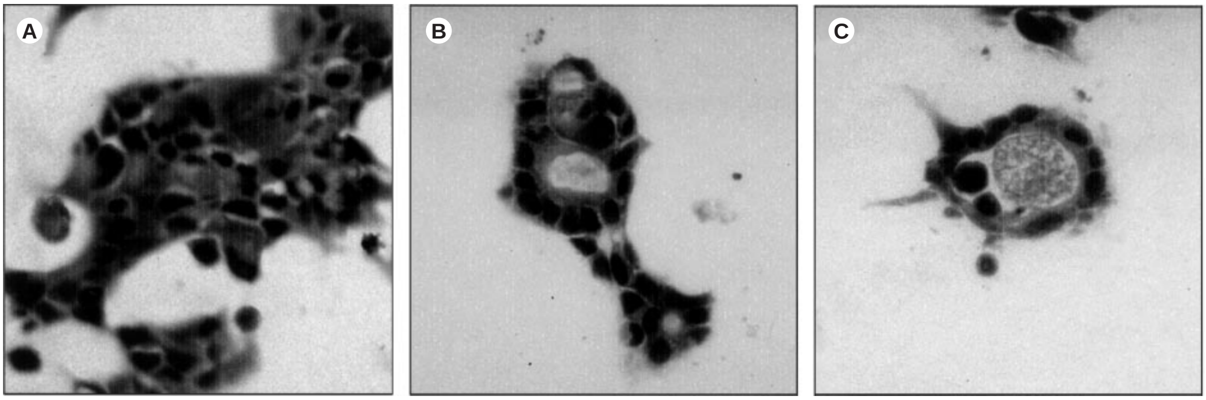

**Figure 3** Morphogenic effect of FGFs on HPAF cells in 3-D collagen gel. Single cell suspension was mixed with neutralised vitrogen solution ($1 \times 10^5$ cells/ml). Cells were fed with standard medium alone **(A)** or supplemented with **(B)** FGF-1 (10 ng/ml) or **(C)** FGF-2 (10 ng/ml), that was replaced every 4 days for 21 days. Experiments were carried out in duplicate and repeated twice

 © 2001 Cancer Research Campaign

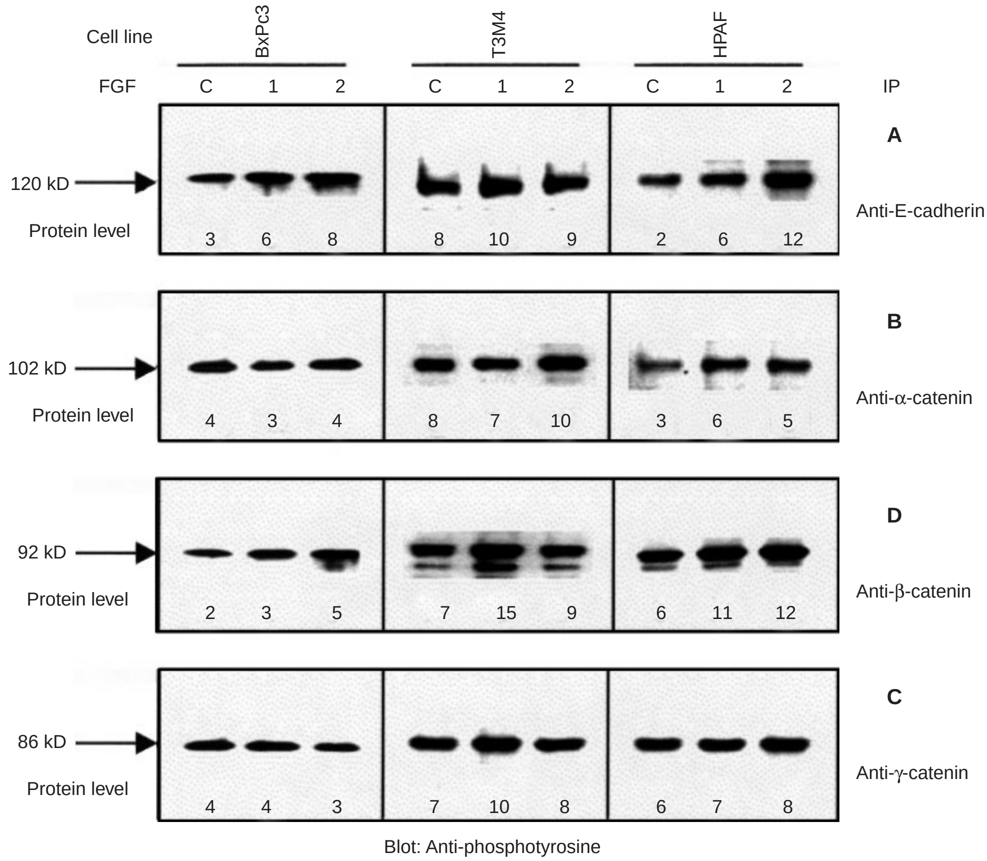

**Figure 4** FGF stimulation of tyrosine phosphorylation of the E-cadherin and catenins in pancreatic cell lines. Serum-starved cell lines were treated with medium alone (C) or with 10 ng/ml FGF-1 (1) or FGF-2 (2) for 15 min at 37˚C, lysed in solubilization buffer containing tyrosine phosphatase inhibitors, and immunoprecipitated with anti-E-cadherin **(A)**, anti-α-catenin **(B)**, anti-β-catenin, **(C)** or anti-γ-catenin **(D)** antibodies. After resolving on SDS-PAGE (8%), proteins were probed with anti-phosphotyrosine antibody. Experiments were repeated twice

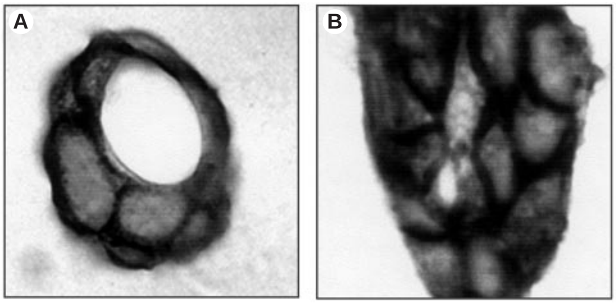

**Figure 5** FGF-2 stimulates membranous localization of E-cadherin **(A)** and β-catenin **(B)** expression in HPAF cells in 3-D collagen gel proteoglycans – in addition to their role in FGF/FGFR system activation – appear to play an important role in maintaining the epithelial phenotype and modulating the expression of E-cadherin and integrins (Day et al, 1999; Kato et al, 1995; Leppa et al, 1996). Taken together, the FGF/FGFR system may influence the differentiation process by regulating the coordinated activities of both cell-cell and cell-matrix machinery.

The present findings are in contrast to the reports suggesting that overexpression of FGFs and FGFRs may be associated with short survival of patients with pancreatic adenocarcinoma (Ohta et al, 1995; Yamanaka et al, 1993). However, it is difficult to draw a firm conclusion in the light of the following observations. The studies to date have included a relatively small number of patients, and it is important to match cases carefully for other factors contributing to survival. Importantly, these studies have shown a correlation between FGF-1 or FGF-2 (with or without coexpression of FGFR) and advanced stage, in no case was the overexpression of FGF and/or FGFR an independent prognostic factor for survival. The nuclear localization of a high molecular weight form of FGF-2 in these studies may also suggest differential effects of FGF-2 in the milieu of the tumour microenvironment. Interestingly, paradoxical findings have been observed in other systems (Blanckaert et al, 1998; McLeskey et al, 1994; Smith et al, 1999; Wang et al, 1997).

The potential involvement of FGF/FGFR in E-cadherin-mediated functions is less well explored. In contrast to our findings, FGF-1 has been found to induce a dysfunctional state of E-cadherin in a rat bladder cell line (Boyer et al, 1992). However, a role for the FGF/FGFR system in the modulation of cell-cell adhesion, invasion and morphogenesis is supported by observations in other systems. The expression of dominant-negative FGFR in PCI2 neuronal cells prevents neurite outgrowth in response to L1, N-CAM or N-cadherin (Saffel et al, 1997). In a recent report, FGF-2 completely inhibited colony formation in soft agar in SK-N-MC cell lines via binding to FGFR, and resulted in the acquisition of a less transformed and more differentiated phenotype (van Puijenbroek et al, 1997).

*© 2001 Cancer Research Campaign*

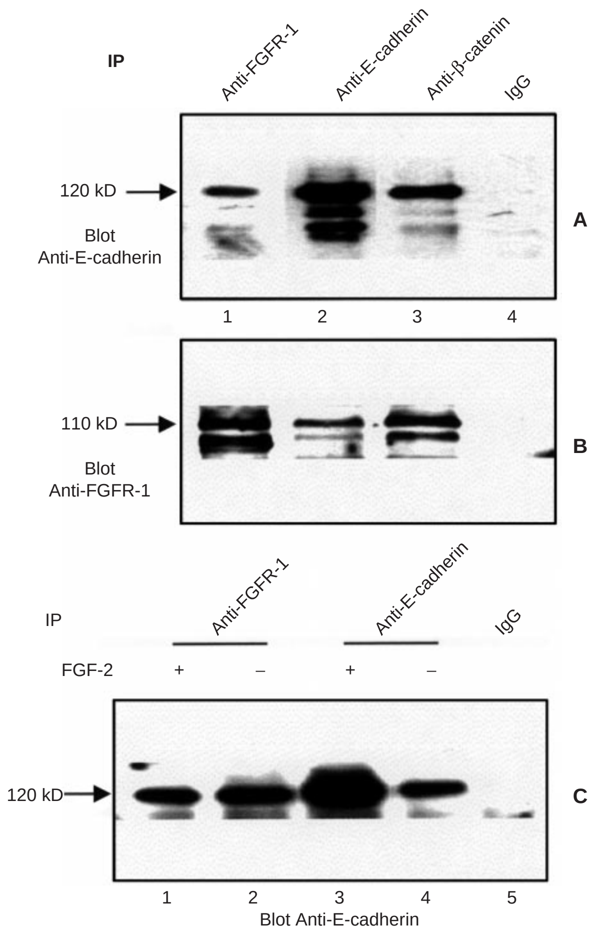

**Figure 6** **(A and B)** Cell extracts from B×Pc3 cell line were immunoprecipitated with anti-FGFR-1 (lane 1), anti-E-cadherin (lane 2), anti-β-catenin (lane 3) antibodies, resolved on 8% polyacrylamide gel, and proteins were transferred onto nitrocellulose membrane. Membranes were then probed with either anti-E-cadherin **(A)** or anti-FGFR-1 **(B)** antibodies. Immunoprecipitates with mouse IgG anti-serum were used as controls for antibody reactivity (lane 4). **(C)** Serum-starved B×Pc3 cells were stimulated with 50 ng/ml FGF-2 for 24 h, and equal aliquots of cell extracts were immunoprecipitated with anti-FGFR-1 (lanes 1 and 2) or anti-E-cadherin (lanes 3 and 4) antibodies. Proteins were separated by SDS-PAGE, transferred onto nitrocellulose membranes, and probed with anti-E-cadherin antibody. Immunoprecipitates with mouse IgG anti-serum were used as controls for antibody reactivity (lane 5). Molecular mass markers are indicated by arrows

The FGFs-induced effects on E-cadherin/catenin function(s) may suggest a post-translational mechanism via modulation of the phosphorylation/dephosphorylation state of one or more components of the cadherin/catenin system. Increased tyrosine phosphorylation of catenins has previously been correlated with the abrogation of cell-cell adhesion (Behrens et al, 1993). Recently however, it has been reported that in primary mouse keratinocytes, β-catenin, γ-catenin and p120$^{ctn}$ become phosphorylated at tyrosine residues upon induction of differentiation with $Ca^{2+}$ treatment (Calautti et al, 1998). These observations suggest that post-translational modification of the E-cadherin system may be cell-type or system-dependent, and hence warrants further investigation.

We have demonstrated a physical association between FGFR-1 and the E-cadherin/catenin system in pancreatic cancer cells. Interestingly, the data suggest that the interaction may primarily involve association between FGFR-1 and β-catenin. Other RTK such as erb-B2 and EGFR have been reported to associate with E-cadherin/catenin system (Hoschuetzky et al, 1994; Kanai et al, 1995). It is possible that FGFR-E-cadherin/catenin interaction facilitates the functional cross-talk between the two systems, and exerts positive regulatory cues as opposed to the negative effects of EGFR.

**Table 1** Effect of FGF-1 and FGF-2 on the expression of E-cadherin and catenins

| Cell lines | BxPc3 | | T3M4 | | HPAF | |
|---|---|---|---|---|---|---|
| | FGF-1 | FGF-2 | FGF-1 | FGF-2 | FGF-1 | FGF-2 |
| *E-cadherin* | | | | | | |
| Expression | + | + | +/– | +/– | + | + |
| Localization | NC | | NC | | M/C→M | |
| *α-catenin* | | | | | | |
| Expression | – | – | – | – | – | – |
| Localization | NC | | NC | | NC | |
| *β-catenin* | | | | | | |
| Expression | +/– | +/– | – | – | +/– | + |
| Localization | NC | | NC | | M/C→M | |
| *γ-catenin* | | | | | | |
| Expression | + | + | –/+ | – | +/– | + |
| Localization | NC | | NC | | NC | |

An avidin-biotin-peroxidase technique was applied. An arbitrary scale was used to evaluate the expression level: -, no difference in expression level compared to untreated cells; +/–, expression is equivocal; and +, increased expression. M/C: mixed membranous and cytoplasmic expression; M: membranous; NC: no change.

### ACKNOWLEDGEMENTS

This work was supported by the Imperial Cancer Research Fund and a Paolo Baffi fellowship (to I. EI-H) from the Fondazione per la Formazione Oncologica (Milan, Italy). The authors thank Dr Helen Hurst for critical reading of the manuscript.

### REFERENCES

Behrens J, Vakaet L, Friis R, Winterhager E, Van Roy F, Mareel MM and Birchmeier W (1993) Loss of epithelial differentiation and gain of invasiveness correlates with tyrosine phosphorylation of the E-cadherin/beta-catenin complex in cells transformed with a temperature-sensitive v-SRC gene. *J Cell Biol* **120**: 757–766

Birchmeier W, Weidner KM and Behrens J (1993) Molecular mechanisms leading to loss of differentiation and gain of invasiveness in epithelial cells. *J Cell Sci Suppl* **17**: 159–164

Blanckaert VD, Hebbar M, Louchez MM, Vilain MO, Schelling ME and Peyrat JP (1998) Basic fibroblast growth factor receptors and their prognostic value in human breast cancer. *Clinical Cancer Research* **4**: 2939–2947

Boyer B, Dufour S and Thiery JP (1992) E-cadherin expression during the acidic FGF-induced dispersion of a rat bladder carcinoma cell line. *Exp Cell Res* **201**: 347–357

Bracke ME, Van Larebeke NA, Vyncke BM and Mareel MM (1991) Retinoic acid modulates both invasion and plasma membrane ruffling of MCF-7 human mammary carcinoma cells in vitro. *Br J Cancer* **63**: 867–872

Bracke ME, Vyncke BM, Bruyneel EA, Vermeulen SJ, De Bruyne GK, Van Larebeke NA, Vleminckx K, Van Roy FM and Mareel MM (1993) Insulin-like growth factor I activates the invasion suppressor function of E-cadherin in MCF-7 human mammary carcinoma cells in vitro. *Br J Cancer* **68**: 282–289

Bracke ME, Charlier C, Bruyneel EA, Labit C, Mareel MM and Castronovo V (1994) Tamoxifen restores the E-cadherin function in human breast cancer MCF-7/6 cells and suppresses their invasive phenotype. *Cancer Res* **54**: 4607–4609

Byers S, Amaya E, Munro S and Blaschuk O (1992) Fibroblast growth factor receptors contain a conserved HAV region common to cadherins and influenza

 © 2001 Cancer Research Campaign strain A hemagglutinins: a role in protein-protein interactions? *Dev Biol* **152**: 411–414

Calautti E, Cabodi S, Stein PL, Hatzfeld M, Kedersha N and Paolo Dotto G (1998) Tyrosine phosphorylation and src family kinases control keratinocyte cell-cell adhesion. *J Cell Biol* **141**: 1449–1465

Day RM, Hao X, Ilyas M, Daszak P, Talbot IC and Forbes A (1999) Changes in the expression of syndecan-1 in the colorectal adenoma-carcinoma sequence. *Virchows Archiv* **434**: 121–125

Doherty P, Williams E and Walsh FS (1995) A soluble chimeric form of the L1 glycoprotein stimulates neurite outgrowth. *Neuron* **14**: 57–66

El-Hariry I, Jordinson M, Lemoine N and Pignatelli M (1999) Characterization of the E-cadherin-catenin complexes in pancreatic carcinoma cell lines. *J Pathol* **188**: 155–162

Hazan RB and Norton L (1998) The epidermal growth factor receptor modulates the interaction of E-cadherin with the actin cytoskeleton. *J Biol Chem* **273**: 9078–9084

Hoschuetzky H, Aberle H and Kemler R (1994) Beta-catenin mediates the interaction of the cadherin-catenin complex with epidermal growth factor receptor. *J Cell Biol* **127**: 1375–1380

Kanai Y, Ochiai A, Shibata T, Oyama T, Ushijima S, Akimoto S and Hirohashi S (1995) c-erbB-2 gene product directly associates with beta-catenin and plakoglobin. *Biochem Biophys Res Commun* **208**: 1067–1072

Kato M, Saunders S, Nguyen H and Bernfield M (1995) Loss of cell surface syndecan-1 causes epithelia to transform into anchorage-independent mesenchyme-like cells. *Mol Biol Cell* **6**: 559–576

Kinoshita Y, Kinoshita C, Heuer JG and Bothwell M (1993) Basic fibroblast growth factor promotes adhesive interactions of neuroepithelial cells from chick neural tube with extracellular matrix proteins in culture. *Development* **119**: 943–956

Leppa S, Vleminckx K, Van Roy F and Jalkanen M (1996) Syndecan-1 expression in mammary epithelial tumor cells is E-cadherin-dependent. *J Cell Sci* **109**: 1393–1403

Leung HY, Gullick WJ and Lemoine NR (1994) Expression and functional activity of fibroblast growth factors and their receptors in human pancreatic cancer. *Int J Cancer* **59**: 667–675

Liu D, Nigam AK, Lalani E-N, Stamp GWH and Pignatelli M (1993) Transfection of E-cadherin into a human colon carcinoma cell line induces differentiation and inhibits growth in vitro. *Gut* **34**: 27

McLeskey SW, Ding IY, Lippman ME and Kern FG (1994) MDA-MB-134 breast carcinoma cells overexpress fibroblast growth factor (FGF) receptors and are growth-inhibited by FGF ligands. *Cancer Res* **54**: 523–530

Metzgar RS, Gaillard MT, Levin SJ, Tuck FL, Bosen EH and Borowitz MJ (1982) Antigens of human pancreatic adenocarcinoma defined by murine monoclonal antibodies. *Cancer Res* **42**: 601–608

Miyamoto S, Teramoto H, Gutkind JS and Yamada KM (1996) Integrins can collaborate with growth factors for phosphorylation of receptor tyrosine kinases and MAP kinase activation: roles of integrin aggregation and occupancy of receptors. *J Cell Biol* **135**: 1633–1642

Ohta T, Yamamoto M, Numata M, Iseki S, Tsukioka Y, Miyashita T, Kayahara M, Nagakawa T, Miyazaki I, Nishikawa K and Yoshitake Y (1995) Expression of basic fibroblast growth factor and its receptor in human pancreatic carcinomas. *Br J Cancer* **72**: 824–831

Okabe T, Yamaguchi N and Ohsawa N (1983) Establishment and characterisation of a carcinoembryonic antigen (CEA)-producing cell line from a human carcinoma of the exocrine pancreas. *Cancer* **51**: 662–668

Pignatelli M, Liu D, Nasim MM, Stamp GW, Hirano S and Takeichi M (1992) Morphoregulatory activities of E-cadherin and beta-1 integrins in colorectal tumour cells. *Br J Cancer* **66**: 629–634

Pignatelli M, Ansari TW, Gunter P, Liu D, Hirano S, Takeichi M, Kloppel G and Lemoine NR (1994) Loss of membranous E-cadherin expression in pancreatic cancer: correlation with lymph node metastasis, high grade, and advanced stage. *J Pathol* **174**: 243–248

Pignatelli M, Karayiannakis AJ, Noda M, Efstathiou J and Kmiot WA (1997) E-cadherin-catenin complex in the gastrointestinal tract. In: *The Gut as a Model in Cell and Molecular Biology*, Halter F, Winton D and Wright NA (eds) pp. 194–203. Kluwer: place of publication.

Roghani M and Moscatelli D (1992) Basic fibroblast growth factor is internalized through both receptor-mediated and heparan sulfate-mediated mechanisms. *J Biol Chem* **267**: 22156–22162

Saffell JL, Williams EJ, Mason IJ, Walsh FS and Doherty P (1997) Expression of a dominant negative FGF receptor inhibits axonal growth and FGF receptor phosphorylation stimulated by CAMs. *Neuron* **18**: 231–242

Smith K, Fox SB, Whitehouse R, Taylor M, Greenall M, Clarke J and Harris AL (1999) Upregulation of basic fibroblast growth factor in breast carcinoma and its relationship to vascular density, oestrogen receptor, epidermal growth factor receptor and survival. *Ann Oncol* **10**: 707–713

Takeichi M (1991) Cadherin cell adhesion receptors as a morphogenetic regulator. *Science* **251**: 1451–1455

Tan MH, Nowak NJ, Loor R, Ochi H, Sandberg AA, Lopez C, Pickren JW, Berjian R, Douglass HO Jr and Chu TM (1986) Characterization of a new primary human pancreatic tumor line. *Cancer Invest* **4**: 15–23

van Puijenbroek AA, van Weering DH, van den Brink CE, Bos JL, van der Saag PT, de Laat SW and den Hertog J (1997) Cell scattering of SK-N-MC neuroepithelioma cells in response to Ret and FGF receptor tyrosine kinase activation is correlated with sustained ERK2 activation. *Oncogene* **14**: 1147–1157

Wang H, Rubin M, Fenig E, DeBlasio A, Mendelsohn J, Yahalom J and Wieder R (1997) Basic fibroblast growth factor causes growth arrest in MCF-7 human breast cancer cells while inducing both mitogenic and inhibitory G1 events. *Cancer Res* **57**: 1750–1757

Watt F and Hertle M (1994) Keratinocyte integrins. In: *The keratinocyte handbook*, Leigh I, Lane B and Watt F (eds) pp 153–164. Cambridge University Press: Cambridge

Williams EJ, Furness J, Walsh FS and Doherty P (1994) Activation of the FGF receptor underlies neurite outgrowth stimulated by L1, N-CAM and N-cadherin. *Neuron* **13**: 583–594

Yamanaka Y, Friess H, Buchler M, Beger HG, Uchida E, Onda M, Kobrin MS and Korc M (1993) Overexpression of acidic and basic fibroblast growth factors in human pancreatic cancer correlates with advanced tumor stage. *Cancer Res* **53**: 5289–5296

© 2001 Cancer Research Campaign 
